# Fecundability in reproductive aged women at risk of sexual dysfunction and associated risk factors: a prospective preconception cohort study

**DOI:** 10.1186/s12884-021-03892-5

**Published:** 2021-06-25

**Authors:** See Ling Loy, Chee Wai Ku, Yin Bun Cheung, Keith M. Godfrey, Yap-Seng Chong, Lynette Pei-Chi Shek, Kok Hian Tan, Fabian Kok Peng Yap, Jonathan Y. Bernard, Helen Chen, Shiao-Yng Chan, Tse Yeun Tan, Jerry Kok Yen Chan

**Affiliations:** 1 Department of Reproductive Medicine, KK Women’s and Children’s Hospital, Singapore, 229899 Singapore; 2 Duke-NUS Medical School, Singapore, 169857 Singapore; 3 Singapore Institute for Clinical Sciences, Agency for Science, Technology and Research (A*STAR), Singapore, 117609 Singapore; 4 Department of Obstetrics & Gynaecology, KK Women’s and Children’s Hospital, Singapore, 229899 Singapore; 5 Program in Health Services & Systems Research and Center for Quantitative Medicine, Duke-NUS Medical School, Singapore, 169857 Singapore; 6 Tampere Center for Child, Adolescent and Maternal Health Research, Tampere University, 33014 Tampere, Finland; 7 Medical Research Council Lifecourse Epidemiology Unit, University of Southampton, Southampton, SO16 6YD UK; 8 National Institute for Health Research Southampton Biomedical Research Centre, University of Southampton and University Hospital Southampton National Health Service Foundation Trust, Southampton, SO16 6YD UK; 9 Yong Loo Lin School of Medicine, National University of Singapore, National University Health System, Singapore, 119228 Singapore; 10 Department of Paediatrics, Yong Loo Lin School of Medicine, National University of Singapore, National University Health System, Singapore, 119228 Singapore; 11 Khoo Teck Puat-National University Children’s Medical Institute, National University Hospital, National University Health System, Singapore, 119074 Singapore; 12 Department of Maternal Fetal Medicine, KK Women’s and Children’s Hospital, Singapore, 229899 Singapore; 13 Department of Paediatrics, KK Women’s and Children’s Hospital, Singapore, 229899 Singapore; 14 Lee Kong Chian School of Medicine, Nanyang Technological University, Singapore, 636921 Singapore; 15 Université de Paris, Centre for Research in Epidemiology and StatisticS (CRESS), Inserm, INRAE, F75004 Paris, France; 16 Department of Psychological Medicine, KK Women’s and Children’s Hospital, Singapore, 229899 Singapore; 17 Department of Obstetrics & Gynaecology, Yong Loo Lin School of Medicine, National University of Singapore, National University Health System, Singapore, 119228 Singapore

**Keywords:** Fecundability, Female sexual function index, Fertility, Preconception, Pregnancy planning, Sexual dysfunction, Time-to-pregnancy

## Abstract

**Background:**

Female sexual dysfunction (FSD) is a prevalent problem, affecting up to 41% of reproductive aged women worldwide. However, the association between female sexual function (FSF) and fecundability in women attempting to conceive remains unclear. We aimed 1) to examine the association between FSF in reproductive-aged preconception Asian women and fecundability, as measured by time-to-pregnancy in menstrual cycles, and 2) to examine lifestyle and behavioral factors associated with FSF.

**Methods:**

From the Singapore PREconception Study of long-Term maternal and child Outcomes (S-PRESTO) prospective cohort, we evaluated FSF using the 6-item Female Sexual Function Index (FSFI-6) and ascertained time-to-pregnancy within a year of baseline assessment. We estimated fecundability ratio (FR) and 95% confidence interval (CI) using the discrete-time proportional hazards model, accounting for left-truncation and right censoring. We used multivariable logistic and linear regression models to identify potential factors related to FSF.

**Results:**

Among 513 participants, 58.9% had low FSF as defined by a total FSFI-6 score at or below the median value of 22. Compared to women with high FSF, those with low FSF had a 27% reduction in fecundability (FR 0.73; 95% CI 0.54, 0.99), with adjustment for age, ethnicity, education, parity and body mass index. Overall, the FRs generally reduced with decreasing FSFI-6 scores. Physical activity, obesity, absence of probable depression and anxiety were independently associated with reduced odds of low FSF and increased FSFI-6 scores, after adjusting for sociodemographic characteristics.

**Conclusions:**

Low FSF is associated with a longer time-to-pregnancy. Early evaluation and optimization of FSF through increased physical activity and optimal mental health may help to improve female fecundity. The finding of obese women having improved FSF remains uncertain which warrants further investigations on plausibly mechanisms. In general, the current finding highlights the importance of addressing FSF in preconception care service for general women, which is currently lacking as part of the fertility promotion effort in the country.

**Supplementary Information:**

The online version contains supplementary material available at 10.1186/s12884-021-03892-5.

## Background

Female sexual dysfunction (FSD) affects many women in the reproductive age group, with an estimated prevalence of 41% worldwide, making it a significant public health problem [1]. According to the American Psychiatric Association’s Diagnostic and Statistical Manual of Mental Disorders (DSM-V), FSD is a heterogeneous combination of disorders that entails sexual interest/ arousal disorder, orgasmic disorder and genito-pelvic pain/ penetration disorder [2]. FSD is multifactorial in etiology, with biological, physical, behavioral and psychosocial issues contributing to the spectrum of FSD [3]. Specifically, factors such as women’s age at marriage, physical health, emotion, exercise, body image, sex education and partner’s sexual health have been consistently associated with female sexual function (FSF) in various populations [3]. Most importantly, impairment in sexual function has been shown to adversely affect a woman’s quality of life and wellbeing [4], with implications for fertility and reproductive health [5].

Sex is a natural and spontaneous expression of intimacy. However, when sex becomes an instrument for conception rather than pleasure, it may create unnecessary pressure and lead to frustration, potentially impeding sexual satisfaction and resulting in a downward spiral [6]. Indeed, situational sexual dysfunction and loss of a couple’s intimacy can happen as a result of timed intercourse around the time of ovulation, or the “fertile period”, where couples only think about conception during intercourse [7]. This is commonly reported by infertile couples who experience infertility-related stress after attempting to conceive for a long period of time, resulting in the need for fertility treatment [7, 8]. There is also evidence showing that attempts to conceive are positively associated with FSF in subfertile women, where the desire for pregnancy could prevail over emotional stress [9]. Taken together, these contradictory findings of the impact of subfertility on FSF may reflect varying responses (i.e. stress vs. desire) of the women while attempting to conceive.

Among women of reproductive age from the general population who are trying to conceive, the association between FSF and fecundability is poorly understood. In particular, it remains unknown to what extent female fecundability (the per-cycle probability of conception) is influenced by FSF. Using data from the Singapore PREconception Study of long-Term maternal and child Outcomes (S-PRESTO) cohort study, we examined FSF and explored its relationship with fecundability as measured by time-to-pregnancy (TTP), in Asian women of reproductive age during the preconception period. We hypothesized that women with low FSF would have a reduced fecundability with longer TTP in 1 year of trying to conceive. In addition, we also examined lifestyle and behavioral factors that could be associated with FSF. Understanding potential risk factors of FSF, particularly modifiable characteristics, may help to devise approaches to reduce FSD, with potential beneficial effect for improved fecundability.

## Materials and methods

### Participants

Data were drawn from the S-PRESTO (ClinicalTrials.gov, NCT03531658), a prospective preconception cohort study that was designed to investigate the long-term influences of events occurring before and during early pregnancy on mother-offspring metabolic and mental health. Asian women of Chinese, Malay or Indian ethnicity attempting to conceive within the next 12 months and aged between 18 and 45 years were enrolled. Ineligibility criteria were known Type 1 diabetes or Type 2 diabetes, had been taking anticonvulsant medication, oral steroids or receiving assisted fertility treatment in the past 1 month. This study was conducted according to the guidelines laid down in the Declaration of Helsinki. The Singhealth Centralized Institute Review Board approved the study protocol (reference 2014/692/D). All participants provided written informed consent.

### Study procedure

Details of the study protocol have been described elsewhere [10]. Briefly, at the recruitment visit (baseline), research staff interviewed women about their sociodemographic characteristics, obstetric histories and lifestyle factors, and performed weight and height measurements in the S-PRESTO cohort center, KK Women’s and Children’s hospital (KKH). At the end of clinic visit, research staff reminded women to perform a pregnancy test using the provided home urinary pregnancy test kits (Biotron Diagnostics, USA) detecting the beta subunit of human chorionic gonadotropin, if their menstrual periods were late for 3–4 days, or 2 weeks after unprotected intercourse. Women were also being reminded to contact the research staff if they had a positive pregnancy test. This was followed by an ultrasound scan to confirm clinical pregnancy. In the absence of any update within 6, 9 and 12 months of recruitment, research staff conducted a follow-up contact by telephone to determine the woman’s pregnancy status. All women were followed for up to 1 year while attempting to conceive.

### Data collection

#### General questionnaire

At baseline, women provided data on date of birth (for age calculation), ethnicity, educational level, parity, menstrual cycle length and regularity, contraception method, date of last menstrual period (LMP) and the number of months of attempting to conceive at study entry. For physical activity assessment, women were asked about their frequency and intensity of physical activity using the short form of the International Physical Activity Questionnaire [11] and were classified into three groups (inactive, minimally active and active) based on the metabolic equivalent task scores in minutes (MET-minutes) [12]. For mental health assessment, women self-administered the Edinburg Postnatal Depression Scale (EPDS) and the state-anxiety subscale of the State Trait Anxiety Inventory (S-STAI), with respective scores of ≥13 indicate probable depression [13] and scores of > 40 indicate probable state anxiety [14, 15]. Although the EPDS has mainly been used in pregnant and postpartum women, it has been found suitable as a depression screener among adults in the community [16]. By calculating the Cronbach’s alpha, high internal consistencies were observed for EPDS (0.83) and S-STAI (0.93) [17].

#### Assessment of FSF

At baseline, women self-administered the 6-item Female Sexual Function Index (FSFI-6) [18], which is a short version of the original 19-item FSFI (FSFI-19) [19]. It is a screening tool that aims to identify women at risk of FSD [18]. The FSFI-6 comprises six questions with each item derived from one of the six domains of the FSFI-19: desire (original item #2), arousal (original item #4), lubrication (original item #7), orgasm (original item #11), satisfaction (original item #16) and pain (original item #17) [18]. Each question provides a score varying from 0 to 5, whose sum yields a total FSFI-6 score ranging from 2 to 30. Higher total score indicates better sexual function. We defined women as low FSF if their total FSFI-6 scores were at or below the median value of 22. This median score approach had been previously adopted by other groups [20, 21]. In this cohort, the internal consistency of the FSFI-6 as measured by Cronbach’s alpha was 0.78.

#### Assessment of TTP

We estimated TTP based on the number of menstrual cycles required to achieve a pregnancy over 12 months of follow-up. We determined pregnancy based on a positive urinary pregnancy test as confirmed by the presence of an intrauterine gestational sac from an ultrasound scan after 6 weeks of gestation. In the event where an ultrasound scan was not available or inconclusive, the diagnosis of pregnancy was made clinically. We calculated the interval between the dates of LMP at recruitment and before conception (for pregnant women) or last follow-up call (for censored women). The interval was converted to cycles by dividing with the average cycle length, which was obtained from the reported minimum and maximum lengths of usual cycles at baseline. TTP was calculated as the total discrete cycles at risk of pregnancy: (days of conception attempt at study entry/ average cycle length) + [(date of LMP before conception or the most recent follow-up) − (date of LMP at recruitment)]/ average cycle length. For women who became pregnant, one more conception cycle was added [10].

#### Statistical analysis

We compared baseline characteristics between women with low and high FSF using the Pearson’s chi-squared test for categorical variables and Mann-Whitney test for continuous variables. We used discrete-time proportional hazards model which analyzed TTP as a discrete scale based on the number of menstrual cycles, to estimate fecundability ratio (FR) and 95% confidence interval (CI) [22, 23], accounting for left truncation and right censoring. The FR represents the cycle-specific probability of conception in one group of women, relative to a control group. A FR < 1 indicates reduced fecundability with longer TTP, while a FR > 1 indicates increased fecundability with shorter TTP. To account for left truncation, we based risk sets only on observed cycles at risk, i.e. cycles of conception attempt while participating in the study. For example, if a woman had been attempting to conceive for two cycles at study entry and reported a pregnancy after six cycles of total attempt time, only four cycles (i.e. 3rd to 6th cycles) as observed in the study contributed to the analysis. Data were censored when women (i) had not conceived after 12 months from the recruitment, (ii) initiated fertility treatment, or (iii) were lost to follow-up or withdrew from the study, whichever occurred first.

Potential confounders that have been commonly reported to influence both FSF and fecundability were determined from the literature [3, 10, 24, 25], based on clinical judgement and using a directed acyclic graph. Those that minimally altered the effect estimates were not included in the final models [26, 27]. The selected potential confounders were age, ethnicity, education, parity and BMI. In view of the possibility that women who had been attempting to conceive for a long period might be suffering from infertility issue, we performed sub-analysis by restricting samples to women who had been attempting conception for ≤12 months at study entry. This would help to exclude potential cases with underlying pathologies in female and male fertility, given that 12-month is a typical length of time after which couples would seek infertility treatment. As FSF might be lower with increasing conception attempts, we performed a sensitivity analysis stratifying on duration attempting to conceive at study entry (dichotomized as ≤6 cycles and ≥ 7 cycles). Owing to the possibility that polycystic ovarian syndrome (PCOS) could affect self-image with potential negative repercussions on sexuality [28], we performed additional analyses excluding women with self-reported PCOS.

We used multivariable logistic and linear regression models to examine potential lifestyle and behavioral factors associated with low FSF (binary dependent variable) and total FSFI-6 scores (continuous dependent variable), respectively. All factors were included simultaneously in the models, except for probable depression and anxiety as both of them were highly correlated with each other (r = 0.74; *p* < 0.001). The models were adjusted for women’s age, ethnicity, education and parity. We used multiple imputation by chained equation to account for missing data, including parity (*n* = 1 woman), BMI (*n* = 3), physical activity (*n* = 2), EPDS scores (*n* = 46) and STAI scores (*n* = 54) [29, 30]. Fifty datasets were generated and the results of the 50 analyses were pooled using Rubin’s rule [31]. We performed sensitivity analyses on the complete-case sample. Statistical analyses were conducted using the SPSS Statistics Version 20 (IBM Corp, Armonk, NY, USA) and Stata Statistical Software, Release 16 (StataCorp, College Station, TX, USA).

## Results

### Study participants

**Fig. 1:**
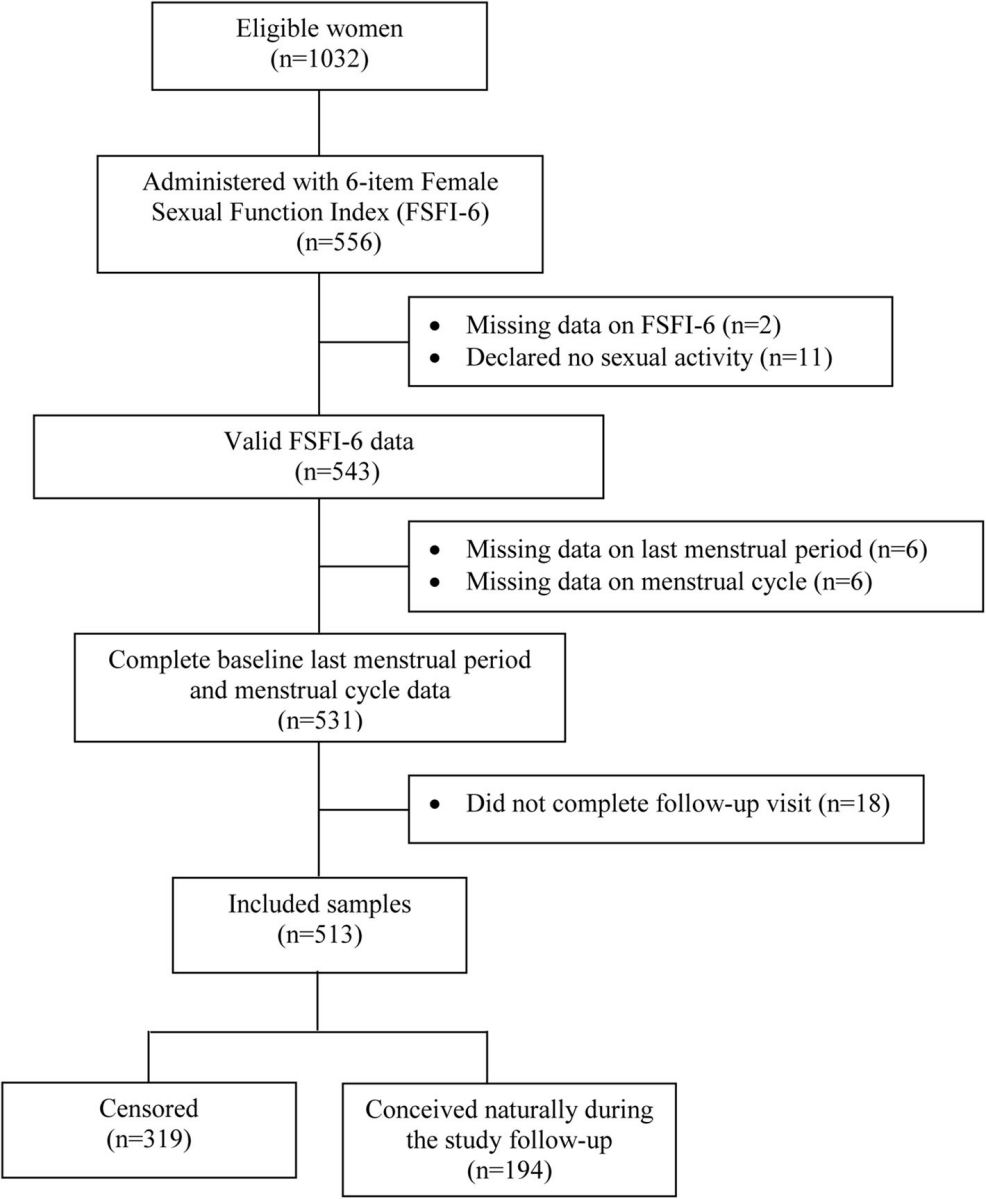
Flowchart showing participants included in the present study

Of 1032 women recruited into the S-PRESTO study, only about the first half of women were invited to complete the FSFI-6. Subsequently, considering overall participant burden and time, the study protocol was modified to reduce measurements and FSFI-6 was removed from the study. As shown in Fig. 1, among 556 women with FSFI-6 administration, 13 were excluded due to incomplete data or reported no sexual activity over the previous 4 weeks (FSFI-6 only captured sexual activities in the past month). Of the remaining 543 women with valid FSFI-6 data, 30 were excluded due to missing and/ or incomplete data for the primary analysis in examining association between FSF and fecundability.

### Characteristics of participants

We included a final sample of 513 women in this study, where 194 women conceived naturally during the 12 months follow-up and 319 women were censored (293 did not conceive, 10 initiated fertility treatment, 16 self-withdrawal). Of these women, 141 (27.5%) achieved a pregnancy within six cycles of follow-up, and 184 (35.9%) within 12 cycles. Compared with excluded women (*n* = 519), included women were similar in ethnicity, parity, BMI, probable depression and anxiety, but older, of lower education and less physically active (see Additional file 1).

Table 1 shows the baseline characteristics of women classified as either non-FSD or probable FSD. Using the FSFI-6 median cut-off score of ≤22, 302 women (58.9%) were found to have low FSF. These women were more likely to be of Chinese ethnicity (80.1% vs. 60.2%), to be underweight (11.6% vs. 6.2%), to be physically inactive (22.8% vs. 18.0%), to exhibit probable depression (17.2% vs. 5.7%) and probable anxiety (25.8% vs. 13.7%), compared to women with high FSF. No differences in age, education, parity, prior use of hormonal contraceptives and amount of time spent trying to conceive at study entry were observed between both groups of women.

**Table 1:** Characteristics between women with low and high FSF from the S-PRESTO study, 2015–2018

	Total (*n* = 513)	Low FSF^a^ (*n* = 302)	High FSF (*n* = 211)
Age, n (%)			
< 35 years	404 (78.8)	236 (78.1)	168 (79.6)
≥ 35 years	109 (21.2)	66 (21.9)	43 (20.4)
Ethnicity, n (%)			
Chinese	369 (71.9)	242 (80.1)	127 (60.2)
Malay	83 (16.2)	37 (12.3)	46 (21.8)
Indian	48 (9.4)	18 (6.0)	30 (14.2)
Mix	13 (2.5)	5 (1.7)	8 (3.8)
Highest education, n (%)			
Primary/ secondary	25 (4.9)	13 (4.3)	12 (5.7)
Post-secondary	189 (36.8)	105 (34.8)	84 (39.8)
Tertiary and above	299 (58.3)	184 (60.9)	115 (54.5)
Parity, n (%)			
0	339 (66.1)	192 (63.6)	147 (69.7)
1	127 (24.8)	80 (26.5)	47 (22.3)
≥ 2	47 (9.2)	30 (9.9)	17 (8.1)
Prior use of hormonal contraceptives in the last 3 months, n (%)			
No	505 (98.4)	300 (99.3)	205 (97.2)
Yes	8 (1.6)	2 (0.7)	6 (2.8)
Body mass index, n (%)			
< 18.5 kg/m^2^	48 (9.4)	35 (11.6)	13 (6.2)
18.5–22.9 kg/m^2^	236 (46.0)	152 (50.3)	84 (39.8)
23–27.4 kg/m^2^	124 (24.2)	67 (22.2)	57 (27.0)
≥ 27.5 kg/m^2^	105 (20.5)	48 (15.9)	57 (27.0)
Physical activity, n (%)			
Inactive	107 (20.9)	69 (22.8)	38 (18.0)
Minimally active	251 (48.9)	154 (51.0)	97 (46.0)
Active	155 (30.2)	79 (26.2)	76 (36.0)
Probable depression, n (%)			
No	449 (87.5)	250 (82.8)	199 (94.3)
Yes	64 (12.5)	52 (17.2)	12 (5.7)
Probable anxiety, n (%)			
No	406 (79.1)	224 (74.2)	182 (86.3)
Yes	107 (20.9)	78 (25.8)	29 (13.7)
Attempted time to conceive at study entry, cycles	4.5 (0.9–11.8)	4.3 (0.9–11.6)	5.0 (1.0–15.5)
≤ 6 cycles	325 (63.4)	201 (66.6)	124 (58.8)
≥ 7 cycles	188 (36.6)	101 (33.4)	87 (41.2)

Values are presented in n (%) for categorical variables and medians (25th – 75th percentiles) for continuous variables. *FSF* female sexual function; *S-PRESTO* Singapore PREconception Study of long-Term maternal and child Outcomes.

^a^Calculated as scores ≤22, the median from the total scores of 6-item Female Sexual Function Index

### Distribution of participants by types of FSD

**Fig. 2:**
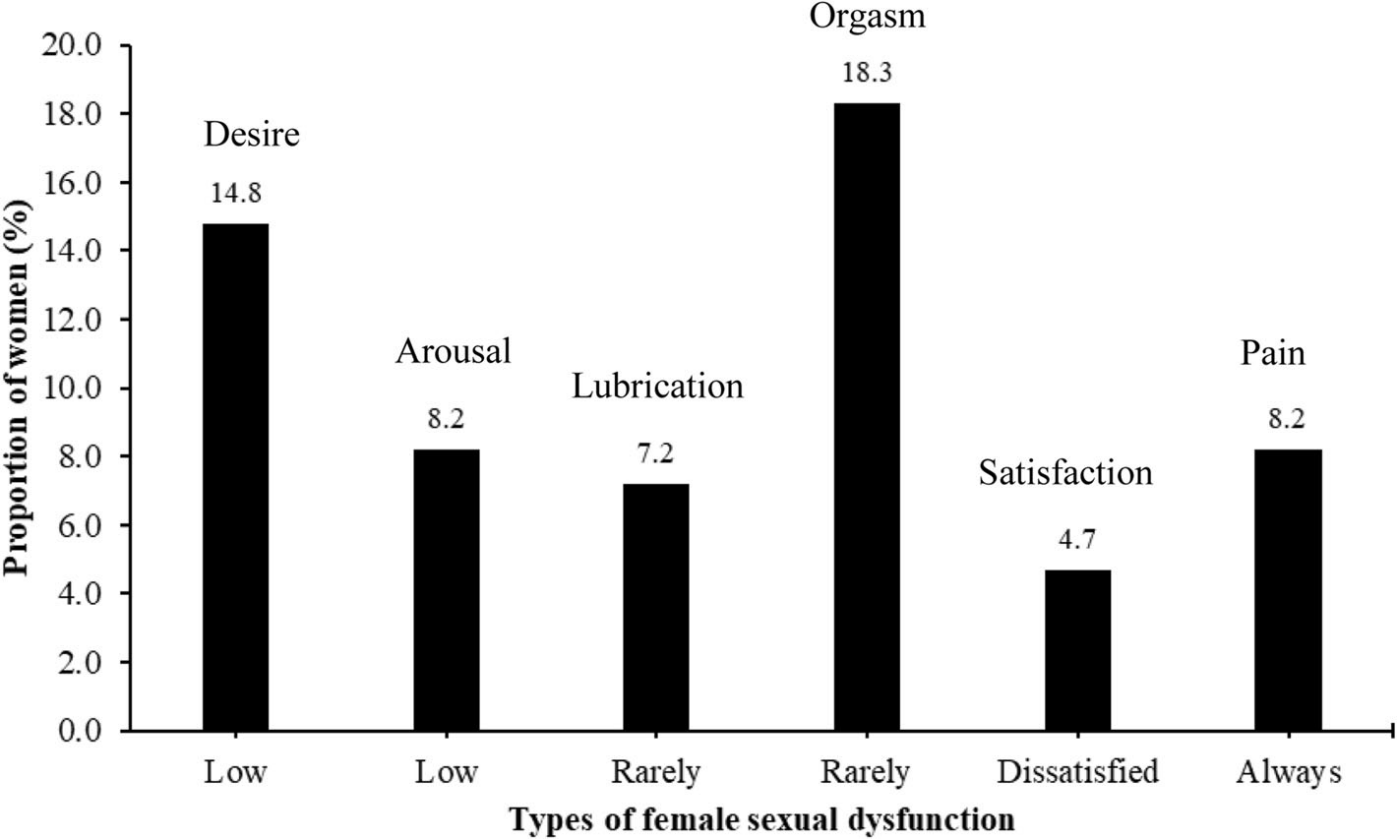
Proportions of women reporting scores ≤2 based on each item of the 6-item Female Sexual Function Index (*n* = 513). Low desire included low, very low or none at all in terms of sexual desire or interest; low arousal included low, very low or none at all in terms of sexual arousal; lubrication rarely included a few times, almost never or never experienced lubrication; rarely orgasm included a few times, almost never or never reach orgasm; dissatisfaction included moderately or very dissatisfied with sexual life; always in pain included most times, almost always or always experienced discomfort or pain during vaginal penetration

Figure 2 presents proportions of women reporting scores ≤2 for each item of FSFI-6. Rarely reaching orgasm (a few times/ almost never/ never; 18.3%) and low sexual desire (low/ very low/ none; 14.8%) were the most frequently reported FSD, followed by low sexual arousal (low/ very low/ none; 8.2%), painful intercourse (most times/ almost always/ always discomfort or pain; 8.2%), rarely experienced sexual lubrication (a few times/ almost never/ never; 7.2%) and sexual dissatisfaction (moderately or very dissatisfied with overall sexual life; 4.7%). Individual scores obtained for each item in the FSFI-6 are presented in Additional file 2.

### Association between FSF and fecundability

**Fig. 3:**
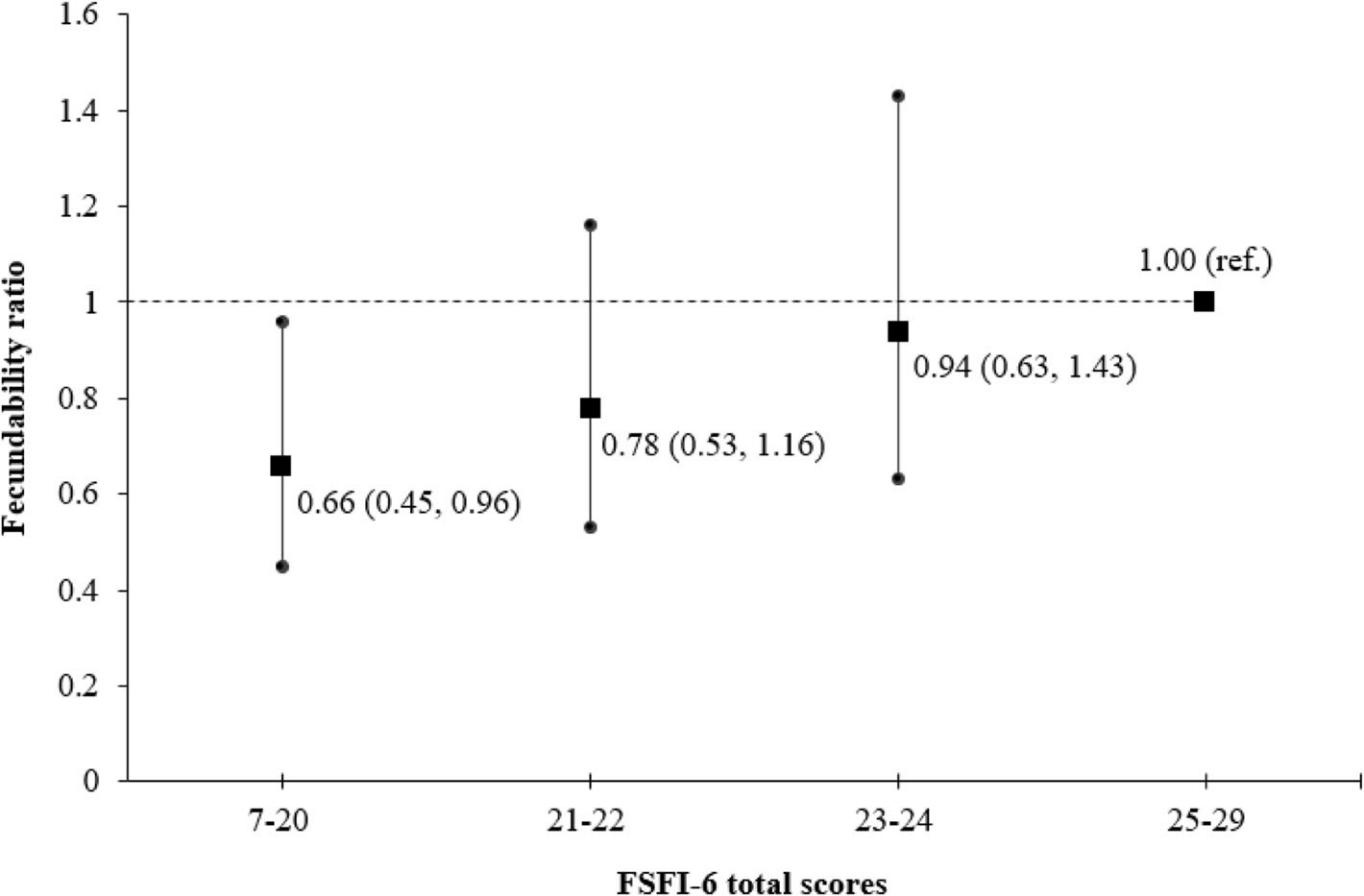
Association between FSFI-6 scores and fecundability. FSFI-6 total scores are divided into quartiles, with the highest quartile as the reference category. The line graph is adjusted for age, ethnicity, education, parity and body mass index. The error bars denote 95% confidence intervals

Table 2 shows the association between low FSF and fecundability as measured by TTP in cycles. Compared to women with high FSF, women with low FSF had a lower FR of 0.73 (95% CI 0.54, 0.99). This association was primarily driven by lower FRs in women reporting lack of lubrication (FR 0.61, 95% CI 0.43, 0.85) and low sexual desire (FR 0.70, 95% CI 0.49, 1.02) (see Additional file 3). Across the range of FSFI-6 total scores, women in the lowest quartile showed the lowest FR of 0.66 (95% CI 0.45, 0.96), compared with women in the highest quartile. Overall, there was a trend of reducing FRs with decreasing FSFI-6 scores (Fig. 3).

**Table 2:** Association between low FSF and fecundability in women from the S-PRESTO study

				Crude model		Adjusted model^a^	
	n	Pregnancies	Cycles	FR	95% CI	FR	95% CI
Low FSF							
Yes	302	109	980	0.82	0.61, 1.09	0.73	0.54, 0.99
No	211	85	866	1.00	(ref.)	1.00	(ref.)

Analyzed using the discrete-time proportional hazards model. *CI* confidence interval; *FR* fecundability ratio; *FSF* female sexual function; *S-PRESTO* Singapore PREconception Study of long-Term maternal and child Outcomes.

^a^Adjusted for age, ethnicity, education, parity and body mass index

**Fig. 4:**
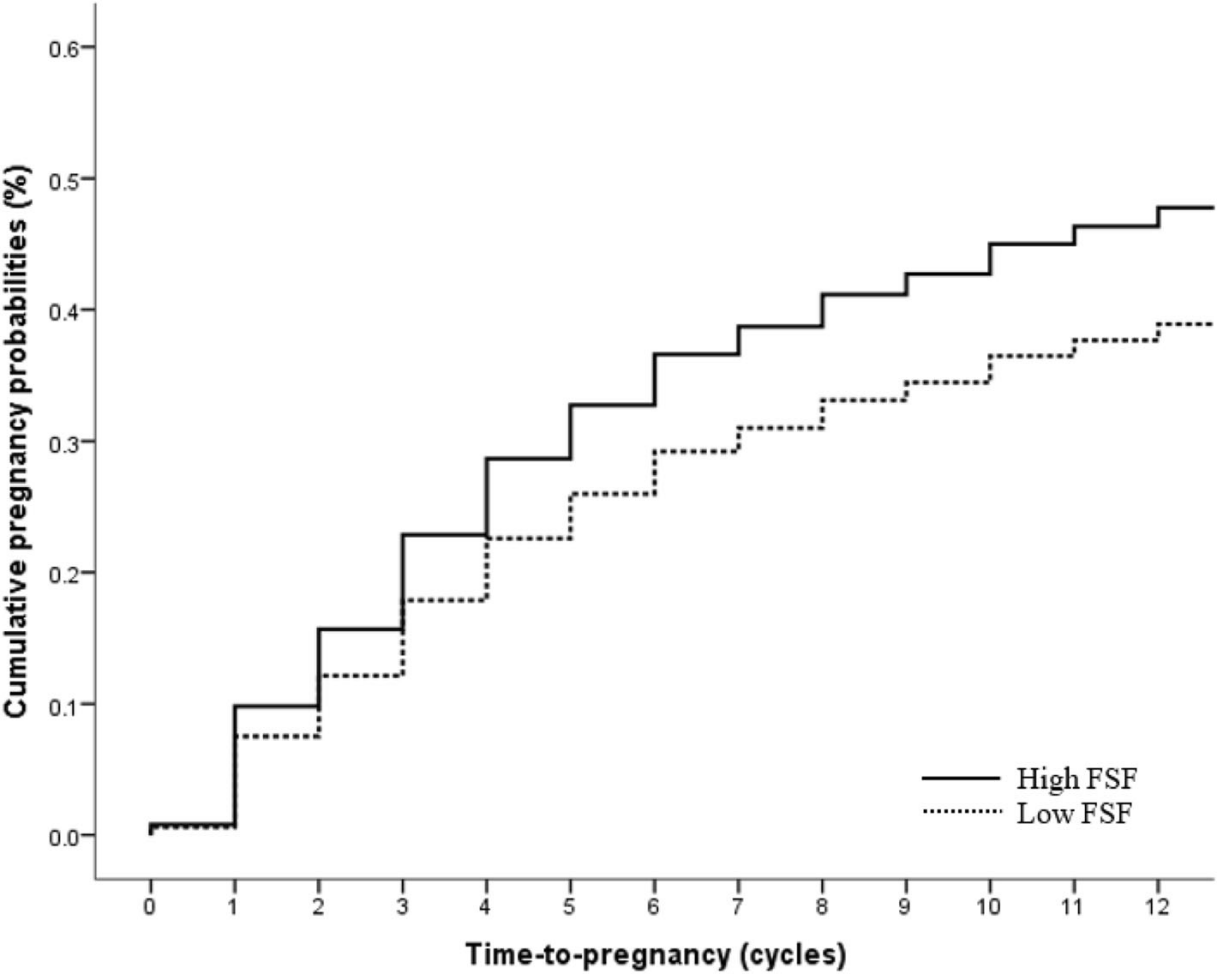
Kaplan-Meier, pregnancy probability curves by female sexual function (FSF) status in women with pregnancy attempt ≤12 months at study entry (n = 390). The curves are adjusted for age, ethnicity, education, parity and body mass index

In the sub-analysis including only women who had been attempting conception for ≤12 months at study entry (*n* = 390), the results were similar where low FSF was still found to have a similarly reduced FR of 0.72 (0.52, 0.99). The Kaplan-Meier curves in Fig. 4 show the cumulative pregnancy probabilities by FSF status in the group of women who had been attempting conception for ≤12 months at study entry. When a sensitivity analysis was performed stratifying on duration attempting to conceive at study entry (dichotomized as ≤6 cycles vs. ≥7 cycles), the association between FSF and fecundability was somewhat stronger in women with ≥7 cycles attempting to conceive at study entry, albeit with overlapping 95% confidence limits (see Additional file 4). When additional analysis was performed by excluding women who reported PCOS (*n* = 7), no substantial change in FR was observed, after adjustment of confounders (0.71; 0.52, 0.95).

### Associations of lifestyle and behavioral factors with FSF

Table 3 shows the potential lifestyle and behavioral factors associated with low FSF and the total FSFI-6 scores. With adjustment for socio-demographic characteristics, being physically active and obesity were associated with reduced odds of low FSF; while probable depression and probable anxiety were associated with increased odds of low FSF. Similar findings were observed when the outcome was based on the total FSFI-6 scores. Inclusion of women with complete dataset in the sensitivity analysis (*n* = 455) revealed similar findings (see Additional file 5).

**Table 3:** Lifestyle and behavioral factors associated with female sexual function in preconception women from the S-PRESTO study

	Low FSF (FSFI-6 scores ≤22)			FSFI-6 scores (continuous)		
Factors	n (%)	OR (95% CI)^a^	OR (95% CI)^b^	mean (SD)	β (95% CI)^a^	β (95% CI)^b^
Physical activity level						
Inactive	69 (22.8)	Reference	Reference	21.08 (3.54)	Reference	Reference
Minimally active	154 (51.0)	0.72 (0.43, 1.19)	0.76 (0.46, 1.26)	21.40 (3.32)	0.51 (−0.23, 1.24)	0.45 (− 0.28, 1.18)
Active	79 (26.2)	0.53 (0.31, 0.92)	0.54 (0.31, 0.93)	22.05 (3.43)	0.87 (0.07, 1.67)	0.87 (0.07, 1.67)
Body mass index					–	
< 18.5 kg/m^2^	35 (11.6)	1.51 (0.74, 3.11)	1.50 (0.73, 3.07)	20.40 (3.75)	−0.70 (−1.70, 0.31)	−0.68 (− 1.68, 0.32)
18.5–22.9 kg/m^2^	152 (50.3)	Reference	Reference	21.13 (3.30)	Reference	Reference
23–27.4 kg/m^2^	67 (22.2)	0.71 (0.44, 1.15)	0.71 (0.44, 1.15)	21.83 (3.57)	0.45 (−0.28, 1.18)	0.45 (−0.28, 1.18)
≥ 27.5 kg/m^2^	48 (15.9)	0.52 (0.29, 0.90)	0.57 (0.33, 0.98)	22.71 (2.84)	1.17 (0.34, 2.01)	1.08 (0.25, 1.91)
Probable depression						
No	250 (82.8)	Reference	–	21.69 (3.38)	Reference	–
Yes	52 (17.2)	4.84 (2.32, 10.13)	–	20.12 (3.67)	−1.92 (−2.81, −1.03)	–
Probable anxiety						
No	224 (74.2)	–	Reference	21.78 (3.28)		Reference
Yes	78 (25.8)	–	2.72 (1.61, 4.59)	20.45 (3.78)	–	−1.65 (−2.36, −0.95)

Associated factors of low FSF (based on FSFI-6 scores ≤22) and total FSFI-6 score (continuous variable) were examined using multivariable logistic and linear regression models, respectively, adjusting for age, ethnicity, education and parity. Probable depression and probable anxiety were not included simultaneously in the models as both variables were highly correlated. Among these women, only three women reported taking anti-depressant and/or anti-anxiety medications. *CI* confidence interval; *FSF* female sexual function; *FSFI-6* 6-item Female Sexual Function Index; *OR* odds ratio; *SD* standard deviation; *S-PRESTO* Singapore PREconception Study of long-Term maternal and child Outcomes.

^a^Model includes physical activity, body mass index and probable depression, but without probable anxiety

^b^Model includes physical activity, body mass index and probable anxiety, but without probable depression

## Discussion

In this prospective preconception cohort study, we examined the association between FSF and fecundability based on TTP among reproductive aged Asian women in Singapore. We found that low FSF, specifically lack of lubrication and low sexual desire, were associated with a reduction in fecundability. In general, worsening FSF as indicated by decreasing FSFI-6 scores was associated with reduced fecundability. This was particularly evident in women who had been attempting to conceive for a longer period at study entry. To investigate potential risk factors of low FSF, we focused on lifestyle and behavioral factors which are modifiable. Women who were physically active and obese were less likely to have low FSF; whereas women with depression and anxiety symptoms were more likely to have low FSF. Taken together, these findings suggest that increasing physical activity and promoting mental wellness may have the potential to improve FSF during the preconception period, leading to shorter TTP and improved fecundability in women of reproductive age. The finding of obese women having improved FSF remains uncertain which warrants further investigations on plausibly mechanisms.

The FSFI-19 is an established instrument used to assess FSF in populations of different ethnicities with good reliability [4]. The Cronbach’s alpha of 0.78 for FSFI-6 in our samples was similar to that presented in the first validation study of the FSFI-6 by Isidori and colleagues [18], reporting Cronbach’s alpha of 0.79. The FSFI-6 was created as a simpler and quicker alternative for easy use in clinical fields and epidemiological studies, with the same psychometric properties [18]. Using the FSFI-6, a cut-off score of ≤19 has been initially proposed to identify women at risk of FSD among Italian women [18]. Considering social and cultural disparities that can influence sexual attitudes [32], studies from other countries have applied different approaches to derive different cut-offs of FSFI-6 to identify high-risk women for FSD [20, 21, 33]. By validating FSFI-6 against FSFI-19, cut-off score of ≤21 was proposed for Korean women [33]. In this study, we utilized a cut-off point based on the FSFI-6 median score of ≤22, similar to the approach used by others for Ecuadorian [20] and Brazilian women [21] with respective ≤20 and ≤ 21 scores reported. Compared to Chedraui et al. [20] and Dall’Agno et al. [21], our women had a slightly higher median score for FSFI-6. This could be explained by the participants almost all being married (98.8%) with presumably stable relationships, potentially placing them in a better psychosocial environment which may contribute to a better sexual function [20, 34].

Nevertheless, to clarify the definite cut-off score for FSD, validation of FSFI-6 against a more accurate test (i.e. sex therapist consultation and physical examination followed by the FSFI-19 assessment) [17] to identify absolute certainty women with and without FSD in our population should be performed. Using the median score approach, we showed that 58.6% of Asian women in Singapore displayed scores ≤22, suggesting at-risk of FSD. Among the six items of sexual disorders as examined in the FSFI-6, low sexual desire and rarely reaching orgasm were more commonly reported in these women. This is consistent with previous reports showing hypoactive sexual desire disorder and orgasmic disorder as major female sexual problems [1, 35]. A study from Malaysia [36], a country that shares many cultural similarities and in close geographical proximity with Singapore, reported that lack of sexual desire was the most common FSD, while lack of sexual satisfaction and lubrication were less commonly reported in Malaysian women, which is in agreement with our findings. Nevertheless, lack of lubrication and low sexual desire were the major FSF issues associated with reduced fecundability among women in this study.

Women with low FSF exhibited a 27% reduction in fecundability compared to women with high FSF, adjusting for confounders. Biologic mechanisms by which FSD could influence fecundability are uncertain. We postulate that the reduced fecundability in women with low FSF may be related to the low coital frequency due to unpleasant sexual experience and difficulties with intimacy [7, 9]. Psychological distress resulting from FSD may also induce an inflammatory response [37], whilst stress-related glucocorticoid changes mediate disruption in reproductive hormonal balance such as luteinizing hormone and melatonin, which in turn can interfere with ovulation [38, 39], contributing to the delayed TTP [24]. In view of the low total fertility rate in Singapore (1.10 in 2020) [40], greater efforts are required with targeted strategies to enhance pregnancy rates. Our findings of the association between FSF and reduced fecundability in preconception women highlights the importance of addressing sexual function in preconception care programs, as part of the fertility promotion effort to increase chances of conception. Importantly, evaluating sexual function during the preconception period provides an opportunity to intervene and resolve sexual dysfunction issues with women and their partners early, resulting in improved preconception quality of life and overall health for the women, with potential beneficial effect on pregnancy outcomes [41].

To manage FSD, it is essential to be aware of related risk factors, in particular those modifiable factors. Consistent with a previous review [3], we observed that women who were physically inactive, and those with depression or anxiety symptoms had a poorer sexual function. An inactive lifestyle and emotional disorder may reduce sexual desire, arousal and satisfaction through disruption of nervous system activity and endocrine factors [42, 43]. Although our study does not demonstrate causal-effect relationships between these factors and FSF, the present findings revealed that physical activity and mental well-being are important lifestyle and behavioral components that must be addressed in sexual health management for preconception women.

To date, the effect of obesity on sexual life in women remains inconclusive. Some studies found no association between BMI and FSF [44, 45]; while others showed increased FSD in overweight and obese women [46–48]. Herein, we observed obese women had improved FSF. We speculate that this may be arisen from the high androgen levels in obese individuals that can increase sexual desire, arousal, orgasm and satisfaction [49]. Indeed, this is supported by Smith et al. [50] demonstrating obese women were more likely than normal weight women to report extreme physical pleasure in sex. The authors pointed out that being obese is not necessarily detrimental to sexual functioning in women [50]. Alternatively, the impact of BMI on coital frequency may account for our result. It is possible that low coital frequency, which has been commonly reported in obese women [51], may translate to a greater desire and arousal, leading to a better FSF. Besides, the present finding could also be partly explained by a lesser degree of obesity (median BMI of 31.3 kg/m^2^ in obese group) in our women, compared with previous reports involving a majority of women from Western clinical setting with a more severe degree of obesity and related comorbid conditions [46, 47, 52]. This is aligned with evidence showing excessive obesity was more likely to be associated with FSD, when different obesity categories were compared [53]. Given that nearly 90% of our women had BMI less than 30 kg/m^2^, obesity-related sex hormone imbalance effects on sexual function may not be readily detectable as the magnitude of FSD may be less pronounced. Overall, these contradictory findings suggest that weight status may not be a good clinical evaluation measure when managing FSD.

There are several limitations that need to be taken into account when interpreting the results. Firstly, FSFI-6 only ascertained sexual activities over the past 4 weeks. Although its validity has not been established in our population, FSFI-6 is proven to have a strong criterion validity across studies and to be a good screening tool for FSD [54]. Secondly, we did not collect information on ovulation, coital timing and frequency throughout the follow-up period to verify the cycles at-risk, which is a limitation of this study. Thirdly, we did not consecutively collect variables which could be varied through the study, such as cycle length, and thus not able to verify the accuracy of self-reported cycle length at baseline. In addition, we did not collect information related to women’s ovarian condition, such as ovarian reserve, which could affect fecundability. Fourthly, we had no information about male partners’ characteristics (e.g. age and education) and sexual functions (e.g. erectile dysfunction and premature ejaculation) which may affect FSF [20, 55].

Finally, the extent to which the present findings could be generalizable to other populations remains to be established, as this study was restricted to planned pregnancies among Asian women in Singapore. Our cohort showed that 35.9% of women spontaneously conceived after 12 cycles of pregnancy attempts, which is lower than the reported rates of 70% or more in some other studies [25, 56, 57]. However, a study of Chinese women had shown similarly a conception rate of 42% after 12 cycles of natural conception [58]. This lower conception rate may help to explain the relatively low total fertility rate in Singapore of 1.10 [40]. In this study, although the recruited women expressed their intention to conceive and were encouraged to engage in sexual intercourse for 2–3 times per week, their frequency of sexual activity might be overestimated, resulting in a low pregnancy rate. Owing to other issues in lifestyle or medical conditions, it was possible that some women might have temporary stopped or delayed their pregnancy attempts during the study without informing the study staff. Additionally, although we excluded women with potential subfertility (conception attempt > 12 months at study entry), we cannot rule out having recruited a group with lower fertility than the general population. Further, differences observed in some characteristics between included and excluded women could potentially reflect an element of selection bias. Nonetheless, this study provides a useful reference for future fertility related studies or family planning interventions for considering FSD assessment and associated factors. This is important as the majority of women are not likely to seek treatment and discuss their sexual problems with physicians unless they are asked [59].

## Conclusions

We observed that low FSF in preconception women of reproductive age is associated with a reduction in fecundability and longer time-to-pregnancy. Early evaluation and optimization of FSF through physical activity and mental wellness promotion in family planning interventions or preconception care can be a pivotal strategy to improve female fecundability. Improving FSF not only helps to shorten TTP, but also improves the overall quality of life in women, resulting in a conducive maternal environment in preparation for pregnancy.

## Supplementary Information

**Additional file 1 **Women’s characteristics according to their inclusion status in the present analysis from the S-PRESTO study (*n* = 1032)

**Additional file 2 **Total and each item scores of the 6-item female sexual function index (*n* = 513).

**Additional file 3.** Associations between types of female sexual function (based on each ≤2 scores in FSFI-6) with fecundability. The line graph is adjusted for age, ethnicity, education, parity and body mass index. The error bars denote 95% confidence intervals. FSFI-6, 6-item Female Sexual Function Index.

**Additional file 4 **Association between female sexual function and fecundability in women from the S-PRESTO study, stratified by cycles of conception attempt at study entry (*n* = 513).

**Additional file 5 **Lifestyle and behavioral factors associated with probable FSD and total FSFI-6 scores in preconception women based on complete dataset (*n* = 455).

## Data Availability

The datasets used and/or analyzed during the current study are available from the corresponding author on reasonable request.

## Abbreviations

BMI: Body mass index
CI: Confidence interval
EPDS: Edinburg Postnatal Depression Scale
FR: Fecundability ratio
FSD: Female sexual dysfunction
FSF: Female sexual function
FSFI-6: 6-item Female Sexual Function Index
FSFI-19: 19-item Female Sexual Function Index
KKH: KK Women’s and Children’s hospital
LMP: Last menstrual period
PCOS: Polycystic ovarian syndrome
S-PRESTO: Singapore PREconception Study of long-Term maternal and child Outcomes
S-STAI: State-anxiety subscale of the State Trait Anxiety Inventory
TTP: Time-to-pregnancy
