# Supplementary material for: Fecundability in reproductive aged women at risk of sexual dysfunction and associated risk factors: a prospective preconception cohort study
Source: BMC Pregnancy Childbirth. 2021 Jun 25;21:444. doi: 10.1186/s12884-021-03892-5 (PMC8228958; doi:10.1186/s12884-021-03892-5)
Supplement: Supplementary file 1 — Additional file 1 Women’s characteristics according to their inclusion status in the present analysis from the S-PRESTO study (n = 1032) [file 12884_2021_3892_MOESM1_ESM.docx]

Fecundability in reproductive aged women at risk of sexual dysfunction and associated risk factors: a prospective preconception cohort study

See Ling Loy, Chee Wai Ku, Yin Bun Cheung, Keith M. Godfrey, Yap-Seng Chong, Lynette Pei-Chi Shek, Kok Hian Tan, Fabian Kok Peng Yap, Jonathan Y. Bernard, Helen Yu Chen, Shiao-Yng Chan, Tse Yeun Tan, Jerry Kok Yen Chan

**Additional file 1:** Women’s characteristics according to their inclusion status in the present analysis from the S-PRESTO study (n=1032).

|  | Included (n=513) | Excluded (n=519) | p^1^ |
| --- | --- | --- | --- |
| Age, n (%) |  |  | <0.001 |
| <35 years | 404 (78.8) | 478 (92.1) |  |
| ≥35 years | 109 (21.2) | 41 (7.9) |  |
| Ethnicity, n (%) |  |  | 0.452 |
| Chinese | 369 (71.9) | 374 (72.1) |  |
| Malay | 83 (16.2) | 76 (14.6) |  |
| Indian | 48 (9.4) | 47 (9.1) |  |
| Mix | 13 (2.5) | 22 (4.2) |  |
| Highest education, n (%) |  |  | 0.011 |
| Primary/ secondary | 25 (4.9) | 28 (5.5) |  |
| Post-secondary | 189 (36.8) | 146 (28.1) |  |
| Tertiary and above | 299 (58.3) | 345 (66.4) |  |
| Parity, n (%) |  |  | 0.303 |
| 0 | 339 (66.1) | 330 (63.6) |  |
| 1 | 127 (24.8) | 149 (28.7) |  |
| ≥2 | 47 (9.2) | 40 (7.7) |  |
| Body mass index, n (%) |  |  | 0.151 |
| <18.5 kg/m^2^ | 48 (9.4) | 38 (7.3) |  |
| 18.5-22.9 kg/m^2^ | 236 (46.0) | 239 (46.2) |  |
| 23-27.4 kg/m^2^ | 124 (24.2) | 152 (29.3) |  |
| ≥27.5 kg/m^2^ | 105 (20.5) | 90 (17.2) |  |
| Physical activity, n (%) |  |  | <0.001 |
| Inactive | 107 (20.9) | 61 (11.8) |  |
| Minimally active | 251 (48.9) | 266 (51.2) |  |
| Highly active | 155 (30.2) | 192 (37.0) |  |
| Probable depression, n (%) |  |  | 0.393 |
| No | 449 (87.5) | 438 (84.4) |  |
| Yes | 64 (12.5) | 81 (15.6) |  |
| Probable anxiety, n (%) |  |  | 0.332 |
| No | 406 (79.1) | 396 (76.3) |  |
| Yes | 107 (20.9) | 123 (23.7) |  |

S-PRESTO, Singapore PREconception Study of long-Term maternal and child Outcomes.

^1^Based on Pearson’s chi-squared test.
