# Supplementary material for: Fecundability in reproductive aged women at risk of sexual dysfunction and associated risk factors: a prospective preconception cohort study
Source: BMC Pregnancy Childbirth. 2021 Jun 25;21:444. doi: 10.1186/s12884-021-03892-5 (PMC8228958; doi:10.1186/s12884-021-03892-5)
Supplement: Supplementary file 2 — Additional file 2 Total and each item scores of the 6-item female sexual function index (n = 513). [file 12884_2021_3892_MOESM2_ESM.docx]

Fecundability in reproductive aged women at risk of sexual dysfunction and associated risk factors: a prospective preconception cohort study

See Ling Loy, Chee Wai Ku, Yin Bun Cheung, Keith M. Godfrey, Yap-Seng Chong, Lynette Pei-Chi Shek, Kok Hian Tan, Fabian Kok Peng Yap, Jonathan Y. Bernard, Helen Yu Chen, Shiao-Yng Chan, Tse Yeun Tan, Jerry Kok Yen Chan

**Additional file 2:** Total and each item scores of the 6-item Female Sexual Function Index (n=513).

| No. | Questions (Over the past 4 weeks…) | Items | Mean | SD | Median | 25^th^ percentile | 75^th^ percentile |
| --- | --- | --- | --- | --- | --- | --- | --- |
| 1 | How would you rate your level (degree) of sexual desire or interest? | Desire | 3.0 | 0.6 | 3.0 | 3.0 | 3.0 |
| 2 | How would you rate your level of sexual arousal (“turn on”) during sexual activity or intercourse? | Arousal | 3.4 | 0.7 | 3.0 | 3.0 | 4.0 |
| 3 | How often did you become lubricated (“wet”) during sexual activity or intercourse? | Lubrication | 3.9 | 1.0 | 4.0 | 3.0 | 5.0 |
| 4 | When you had sexual stimulation or intercourse, how often did you reach orgasm? | Orgasm | 3.4 | 1.1 | 3.0 | 3.0 | 4.0 |
| 5 | How satisfied have you been with your overall sexual life? | Satisfaction | 3.9 | 0.8 | 4.0 | 4.0 | 4.0 |
| 6 | How often did you experience discomfort or pain during vaginal penetration? | Pain | 3.9 | 1.0 | 4.0 | 3.0 | 5.0 |
|  |  | Total | 21.5 | 3.4 | 22.0 | 19.0 | 24.0 |

SD, standard deviation.
