# Supplementary material for: Fecundability in reproductive aged women at risk of sexual dysfunction and associated risk factors: a prospective preconception cohort study
Source: BMC Pregnancy Childbirth. 2021 Jun 25;21:444. doi: 10.1186/s12884-021-03892-5 (PMC8228958; doi:10.1186/s12884-021-03892-5)

Fecundability in reproductive aged women at risk of sexual dysfunction and associated risk factors: a prospective preconception cohort study

See Ling Loy, Chee Wai Ku, Yin Bun Cheung, Keith M. Godfrey, Yap-Seng Chong, Lynette Pei-Chi Shek, Kok Hian Tan, Fabian Kok Peng Yap, Jonathan Y. Bernard, Helen Yu Chen, Shiao-Yng Chan, Tse Yeun Tan, Jerry Kok Yen Chan

**Additional file 3:** Associations between types of female sexual function (based on each ≤2 scores in FSFI-6) with fecundability. The line graph is adjusted for age, ethnicity, education, parity and body mass index. The error bars denote 95% confidence intervals. FSFI-6, 6-item Female Sexual Function Index.


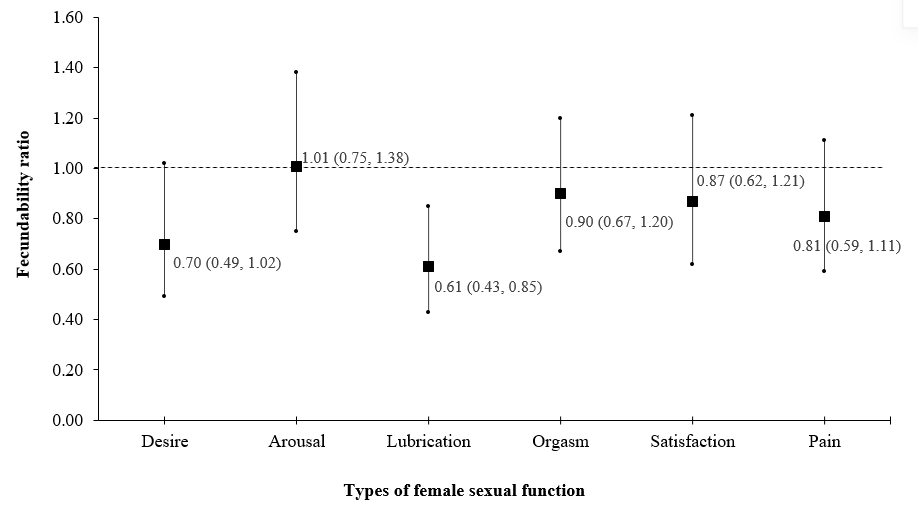

Supplement: Supplementary file 3 — Additional file 3. Associations between types of female sexual function (based on each ≤2 scores in FSFI-6) with fecundability. The line graph is adjusted for age, ethnicity, education, parity and body mass index. The error bars denote 95% confidence intervals. FSFI-6, 6-item Female Sexual Function Index. [file 12884_2021_3892_MOESM3_ESM.docx]
