# Supplementary material for: Fecundability in reproductive aged women at risk of sexual dysfunction and associated risk factors: a prospective preconception cohort study
Source: BMC Pregnancy Childbirth. 2021 Jun 25;21:444. doi: 10.1186/s12884-021-03892-5 (PMC8228958; doi:10.1186/s12884-021-03892-5)
Supplement: Supplementary file 4 — Additional file 4 Association between female sexual function and fecundability in women from the S-PRESTO study, stratified by cycles of conception attempt at study entry (n = 513). [file 12884_2021_3892_MOESM4_ESM.docx]

Fecundability in reproductive aged women at risk of sexual dysfunction and associated risk factors: a prospective preconception cohort study

See Ling Loy, Chee Wai Ku, Yin Bun Cheung, Keith M. Godfrey, Yap-Seng Chong, Lynette Pei-Chi Shek, Kok Hian Tan, Fabian Kok Peng Yap, Jonathan Y. Bernard, Helen Yu Chen, Shiao-Yng Chan, Tse Yeun Tan, Jerry Kok Yen Chan

**Additional file 4:** Association between female sexual function and fecundability in women from the S-PRESTO study, stratified by cycles of conception attempt at study entry (n=513).

|  | Cycles of conception attempt at study entry | | | | |
| --- | --- | --- | --- | --- | --- |
| FSFI-6 total scores | ≤6 cycles (n=325) | |  | ≥7 cycles (n=188) | |
|  | FR | 95% CI |  | FR | 95% CI |
| FSFI-6 scores (median) |  |  |  |  |  |
| Low (7-22) | 0.76 | 0.54, 1.06 |  | 0.64 | 0.31, 1.34 |
| High (23-29) | 1.00 | (ref.) |  | 1.00 | (ref.) |
|  |  |  |  |  |  |
| FSFI-6 scores (quartile) |  |  |  |  |  |
| Q1 (7-20) | 0.72 | 0.45, 1.14 |  | 0.42 | 0.13, 1.35 |
| Q2 (21-22) | 0.77 | 0.47, 1.26 |  | 0.74 | 0.26, 2.10 |
| Q3 (23-24) | 0.95 | 0.57, 1.59 |  | 0.84 | 0.32, 2.23 |
| Q4 (25-29) | 1.00 | (ref.) |  | 1.00 | (ref.) |

Analyzed using the discrete-time proportional hazards model. CI, confidence interval; FR, fecundability ratio; FSFI-6, 6-item Female Sexual Function Index; S-PRESTO, Singapore PREconception Study of long-Term maternal and child Outcomes. Models are adjusted for age, ethnicity, education, parity and body mass index.
