# Supplementary material for: Fecundability in reproductive aged women at risk of sexual dysfunction and associated risk factors: a prospective preconception cohort study
Source: BMC Pregnancy Childbirth. 2021 Jun 25;21:444. doi: 10.1186/s12884-021-03892-5 (PMC8228958; doi:10.1186/s12884-021-03892-5)
Supplement: Supplementary file 5 — Additional file 5 Lifestyle and behavioral factors associated with probable FSD and total FSFI-6 scores in preconception women based on complete dataset (n = 455). [file 12884_2021_3892_MOESM5_ESM.docx]

Fecundability in reproductive aged women at risk of sexual dysfunction and associated risk factors: a prospective preconception cohort study

See Ling Loy, Chee Wai Ku, Yin Bun Cheung, Keith M. Godfrey, Yap-Seng Chong, Lynette Pei-Chi Shek, Kok Hian Tan, Fabian Kok Peng Yap, Jonathan Y. Bernard, Helen Yu Chen, Shiao-Yng Chan, Tse Yeun Tan, Jerry Kok Yen Chan

**Additional file 5:** Lifestyle and behavioral factors associated with low FSF and total FSFI-6 scores in preconception women based on complete dataset (n=455).

|  | Low FSF (FSFI-6 scores ≤22) | |  | FSFI-6 scores (continuous) | |
| --- | --- | --- | --- | --- | --- |
| Factors | OR (95% CI) | OR (95% CI) |  | β (95% CI) | β (95% CI) |
| Physical activity level |  |  |  |  |  |
| Inactive | Reference | Reference |  | Reference | Reference |
| Minimally active | 0.72 (0.42, 1.22) | 0.76 (0.45, 1.29) |  | 0.58 (-0.19, 1.34) | 0.51 (-0.25, 1.27) |
| Active | 0.49 (0.28, 0.88) | 0.50 (0.28, 0.88) |  | 1.09 (0.24, 1.93) | 1.08 (0.24, 1.92) |
| Body mass index |  |  |  | - |  |
| <18.5 kg/m^2^ | 1.57 (0.72, 3.42) | 1.53 (0.70, 3.34) |  | -0.74 (-1.82, 0.34) | -0.71 (-1.78, 0.37) |
| 18.5-22.9 kg/m^2^ | Reference | Reference |  | Reference | Reference |
| 23-27.4 kg/m^2^ | 0.71 (0.43, 1.17) | 0.71 (0.42, 1.17) |  | 0.38 (-0.38, 1.14) | 0.38 (-0.37, 1.14) |
| ≥27.5 kg/m^2^ | 0.45 (0.25, 0.81) | 0.49 (0.28, 0.88) |  | 1.23 (0.35, 2.10) | 1.14 (0.27, 2.01) |
| Probable depression |  |  |  |  |  |
| No | Reference | - |  | Reference | - |
| Yes | 4.57 (2.16, 9.64) | - |  | -1.78 (-2.70, -0.87) | - |
| Probable anxiety |  |  |  |  |  |
| No | - | Reference |  |  | Reference |
| Yes | - | 2.63 (1.54, 4.50) |  | - | -1.59 (-2.32, -0.86) |

Associated factors of low FSF (based on score ≤22 FSFI-6) and total FSFI-6 score (continuous variable) were examined using multiple logistic and multiple linear regression models, respectively, adjusting for age, ethnicity, education and parity. Probable depression and probable anxiety were not included simultaneously in the models as both variables were highly correlated. CI, confidence interval; FSF, female sexual function; FSFI-6, 6-item Female Sexual Function Index; OR, odds ratio.
